# Critical evaluation of a putative glucosamine excretion by *Aspergillus niger* CBS120.49 and *Penicillium ochrochloron* CBS123.824 under citric acid producing conditions

**DOI:** 10.1038/s41598-019-43976-z

**Published:** 2019-05-16

**Authors:** Desirée Josefine Artmann, Werner Amrain, Adele Murauer, Markus Ganzera, Pamela Vrabl, Christoph Walter Schinagl, Wolfgang Burgstaller

**Affiliations:** 1 Institute of Microbiology, University of Innsbruck, Technikerstrasse 25, 6020 Innsbruck, Austria; 2 Institute of Pharmacy, Pharmacognosy, University of Innsbruck, Innrain 80-82, 6020 Innsbruck, Austria

**Keywords:** Metabolomics, Fungal physiology

## Abstract

As one of the most frequently occurring monomers in the biosphere, glucosamine is a valuable metabolite for several applications. Although microbial glucosamine production is still in its infancy, it offers the possibility to circumvent problems associated with traditional production by hydrolysis. Of particular interest is a study with *Aspergillus niger*, which reports for the first time high glucosamine excretion in the early phase of citric acid production. These results have relevance for both the commercial glucosamine production and deeper insight into the regulation of organic acid excretion in fungi. To investigate glucosamine excretion, we performed bioreactor batch cultivations with *Penicillium ochrochloron* CBS123.824 and *A*. *niger* CBS120.49 using cultivation conditions which are known to trigger the production of citric acid. Glucosamine detection in culture filtrates was achieved by two photometric methods, High performance liquid chromatography with evaporative light scattering detection (HPLC-ELSD) and HPLC with mass spectrometry detection (HPLC-MS). Surprisingly, we detected no glucosamine at all. Based on a critical review of published data for *A*. *niger*, we conclude that the reported high levels of excreted glucosamine might be an experimental artifact. However, growth experiments with glucosamine as a combined or single source for carbon or nitrogen showed that both organisms are in principle able to transport glucosamine across their plasma membrane, which is a prerequisite for the excretion of glucosamine.

## Introduction

As a major component of chitin and chitosan in the exoskeleton of Arthropoda and in the cell walls of fungi, glucosamine is one of the most abundant monomers in the biosphere^1,2^. The global sales volume of glucosamine, which is widely used in medical applications and in the food and cosmetics industry^3,4^, has been estimated at $2 billion^5^ and is still rising.

One problem is, that glucosamine bound in chitin or chitosan is not readily accessible: Currently, most glucosamine is produced by acidic or enzymatic hydrolysis of chitin from shrimp shells or crabs^6,7^. Glucosamine production also faces some problems as raw materials^8^ and enzymes^9^ are limited and some people show allergic reactions to shellfish^10^. In the last decade more attention has been paid to the microbial production of glucosamine as it has the potential to circumvent several problems of traditional production techniques^5^. In principle, microbial glucosamine production follows two different strategies: The first strategy - mainly performed with fungi such as *Rhizopus oligosporus*, *Monascus pilosus* and *Aspergillus* sp.^5,11^ - aims to increase the glucosamine content of the cell wall by optimising different culture conditions such as the availability of dissolved oxygen or the addition of stimulating factors such as methanol^5^. The glucosamine concentration achieved with this strategy was in the range of 0.1–14.4 g/L glucosamine^5,6,12–18^ (Table 1) and therefore still insufficient. In contrast, the second strategy, using metabolically engineered *Escherichia coli* or *Bacillus subtilis*, aims to induce the excretion of glucosamine and N-acetyl glucosamine^5^. This strategy was reported to achieve N-acetyl glucosamine concentrations in the culture broth of up to 110 g/L^19^. However, the underlying regulation mechanisms of glucosamine excretion are largely unknown^5^.

**Table 1:** Overview of glucosamine concentrations achieved with different fungi.

Fungus	Glucosamine concentration	Glucosamine localisation	Reference
*Monascus pilosus* KCCM 60029 (ATCC 22080)	0.264 g/L	fungal cell wall	^12^
*Monascus pilosus* BCRC 31527	0.719 g/L	fungal cell wall	^13^
*Rhizopus oligosporus* NRRL 2710	0.107 g/g biomass	fungal cell wall	^14^
*Rhizopus oligosporus* BCRC 31996	0.394 g/L	fungal cell wall	^13^
*Aspergillus sp*. BCRC 31742	3.428 g/L	fungal cell wall	^13^
*Aspergillus sp*. BCRC 31742	7.05 g/L	fungal cell wall	^6^
	7.48 g/L (methanol addition)		
*Aspergillus sp*. BCRC 31742	5.48 g/L (flasks)	fungal cell wall	^15^
	3.91 g/L (fermenter)		
*Aspergillus sp*. BCRC 31742	14.37 g/L	fungal cell wall	^16^
*Monascus indicus* CCUG 22424	0.37 g/L	fungal cell wall	^17^
*Actinomucor elegansCGMCC3539*	5.12 g/L	fungal cell wall	^18^
	6.85 g/L (methanol addition)		
*Aspergillus niger* PM1	48 g/L	extracellular	^20^
*Aspergillus niger* PM1	46 g/L	extracellular	^21^

However, in fungi the highest yield reported (i) for elevated amounts of glucosamine embedded in the biomass was 14 g/L^16^ and (ii) for excreted glucosamine was 48 g/L^20,21^. In detail, these two reports^20,21^ state for *Aspergillus niger* PM1 an excretion of the above mentioned 48 g/L glucosamine in the early phase (15–55 h) of citric acid production (Table 1). Further, glucose and ammonium were proposed to be taken up separately by the fungus and glucosamine would then be excreted into the medium^20,21^ and taken up again during later cultivation phases. Another report^22^ described for *A*. *niger* the presence of a sticky, low molecular weight compound under conditions of organic acid production. This compound was also reported being excreted and reutilised during later phases of cultivation. The reported high levels of excreted glucosamine from *A*. *niger* PM1 are of multiple interest. Firstly, the isolation of excreted glucosamine would be technically and economically much more favorable, especially if compared to the traditional production of glucosamine, which involves several steps to gain access to the glucosamine bound in chitin or chitinosan^11^. Secondly, the excretion and reutilisation of glucosamine in general, would represent an important and so far unexplored path of the physiological network. Such a metabolic path could contribute to a “hidden” (caused by excretion and reutilisation) diauxic growth induced by the sequential use of the carbon/energy sources glucose and glucosamine^23^. This would be important to further elucidate the regulation of organic acid excretion in fungi.

In our studies on organic acid excretion by *Penicillium ochrochloron* we often observed the following: strong filter clogging when filtering samples only during a short period of cultivation (e.g. ref.^24^), which might have been the consequence of a compound excreted and afterwards taken up again. Also unidentified excreted compounds were detected in high performance liquid chromatography (HPLC) chromatograms of culture broths. Due to the reported high glucosamine excretion by *A*. *niger*, we wondered whether one of these unidentified compounds excreted by *P*. *ochrochloron* could be glucosamine. We therefore investigated glucosamine excretion in *P*. *ochrochloron* CBS123.824 using standard cultivation conditions of our own work and that of *A*. *niger*^20^, and for comparison in *A*. *niger* using the originally reported cultivation conditions^20^. Unfortunately, *A*. *niger* PM1 was no longer available (information from two authors from ref.^20^). Instead, we used *A*. *niger* CBS120.49 (=*A*. *niger* A158), a strain hypothesised to excrete glucosamine in the early stages of cultivation under citric acid excretion conditions^25^.

In brief, glucosamine could not be detected in culture broths of *P*. *ochrochloron* or *A*. *niger*, despite the application of four different analytical methods: two photometric methods, and two high performance liquid chromatography methods, one with an evaporating light scattering detector (HPLC-ELSD) and the other with a mass spectrometer as detector (HPLC-MS). Based on the results of this intensive analytical investigation, including a critical review of glucosamine excretion by *A*. *niger*^20^ we conclude that the reported high levels of excreted glucosamine are most likely an experimental artifact. However, growth experiments with glucosamine as a combined or single C or N source showed that both *P*. *ochrochloron* and *A*. *niger*, are in principle able to transport this compound across their plasma membrane, which is a prerequisite for glucosamine excretion.

## Results

### Media and growth characteristics

The starting point for this study were the cultivation conditions, which were claimed to trigger glucosamine excretion in *A*. *niger* PM1^20^. These cultivation conditions, which also triggered enhanced citric acid excretion in this organism, are characterised by a pH of 2.1, a high glucose concentration (833 mM) and early depletion of ammonium (initial concentration 38 mM; exhaustion between 40 and 50 h of cultivation). Since the reported excretion and re-utilisation of glucosamine might represent an important and so far unexplored physiological process during the early stages of citric acid production, special emphasis was laid upon this cultivation phase.

**Figure 1:**
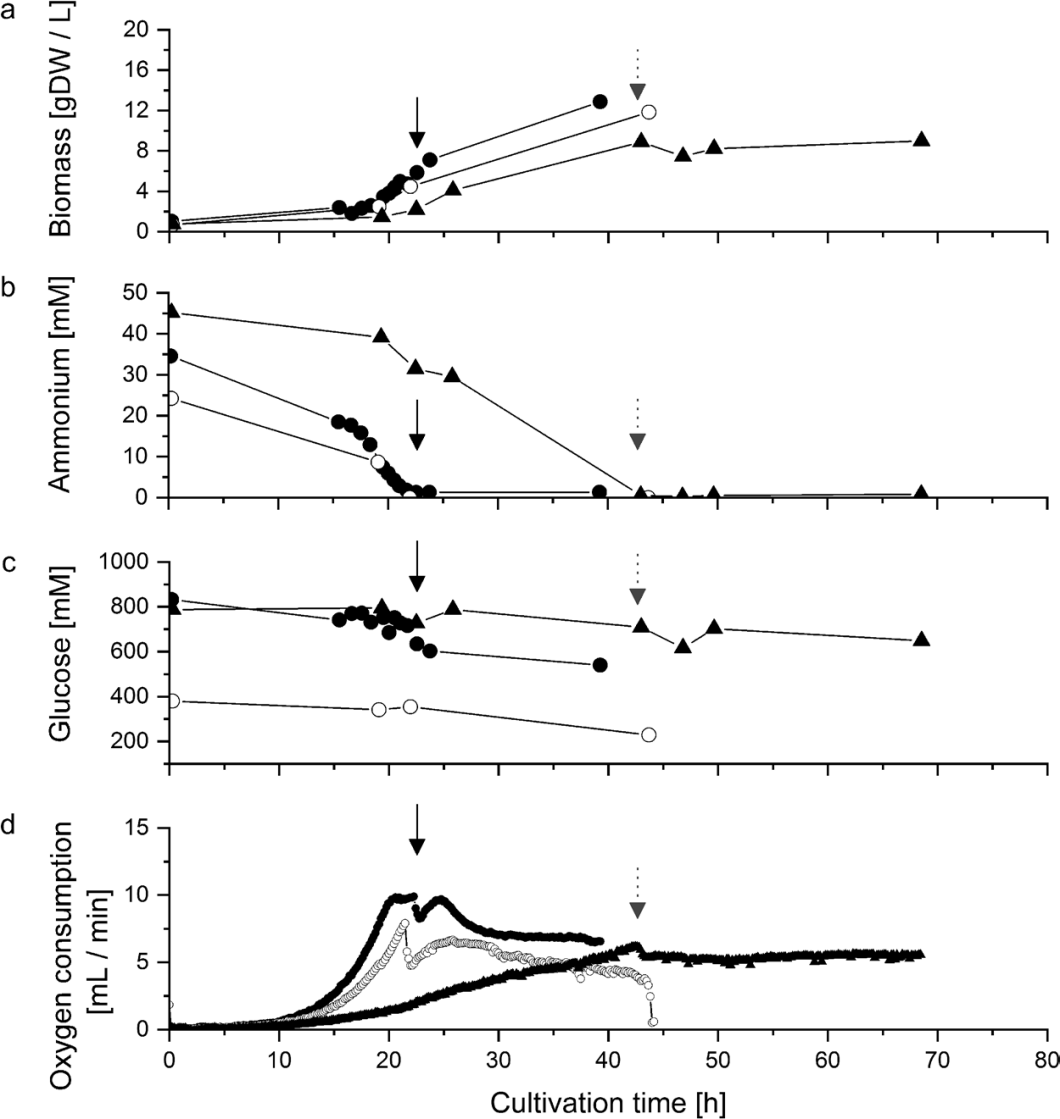
Growth characteristics of bioreactor batch cultures of *Penicillium ochrochloron* CBS123.824 and *Aspergillus niger* CBS120.49. (**a**) Biomass formation, (**b**) ammonium concentration, (**c**) glucose concentration and (**d**) oxygen consumption. Black arrows denote the time point of ammonium exhaustion in both *P*. *ochrochloron* cultures, dashed grey arrows in *A*. *niger* cultures. (▲) *A*. *niger*, cultivation conditions after ref.^20^. (●) *P*. *ochrochloron* cultivation conditions after ref.^20^. (○) *P*. *ochrochloron* in ammonium limited standard medium after ref.^26^. Duplicate experiments are shown in Supplementary Fig. S1.

To explore this issue, we cultivated *A*. *niger* CBS120.49 according to the published conditions^20^ except the bioreactor equipment. Indeed, the resulting nutrient uptake pattern, growth characteristics and citrate excretion of *A*. *niger* CBS120.49 (Figs 1, S1) were comparable to those reported^20^ (Table 2), indicating that this strain was a suitable alternative to the originally used but no longer available *A*. *niger* PM1 strain. The non-exponential respiration rates of *A*. *niger* CBS120.49 (Figs 1, S1) and the considerably low concentration of trace elements in this medium^20^ indicated that the culture was already limited with respect to trace-elements prior to the exhaustion of ammonium in the medium.

**Table 2:** Growth characteristics of bioreactor batch grown *Aspergillus niger* PM1, *A*. *niger* CBS120.49 and *Penicillium ochrochloron* CBS123.824. Data for *A*. *niger* PM1, a strain which was reported to excrete high amounts of glucosamine, were derived and extrapolated from an earlier published report^20^.

Organism	*Aspergillus niger*		*Penicillium ochrochloron*	
Strain	PM1	CBS120.49	CBS123.824	CBS123.824
Growth conditions according to reference	(20)	(20)	(20)	(26)
	original work	present work	present work	present work
Glucose concentration in the medium	150 g	150 g	150 g	80.85 g
Time of NH_4_ exhaustion (h), i.e. end of exponential growth	44	42.5	22	22
Citrate at 44 h [mM]	5	5	—	2
Lag phase (h) calculated from biomass	14	15	14	13
Lag phase (h) calculated from oxygen consumption	—	5	8	5
Biomass at 22 h [g DW L^−1^]	1	2	6	5

Since *P*. *ochrochloron* differed in its conditions needed for an enhanced citric acid excretion (pH 7; composition of trace element solution), the original cultivation conditions for *A*. *niger*^20^ were slightly modified: this included increasing the cultivation pH from 2.1^20^ to 7 and using our standard trace element solution for this organism. For comparison, *P*. *ochrochloron* was also grown in our standard growth medium, where ammonium is the first nutrient to be depleted^26^. Both *P*. *ochrochloron* cultivation conditions triggered typical ammonium limited growth characteristics with a distinct exponential growth phase, which was mirrored in the respective respiration rates (Figs 1, S1).

### Method development for glucosamine analysis in culture broths

Extracellular glucosamine concentrations were determined by four different analytical approaches. Since glucosamine was reported to be excreted in very high amounts up to 48 g/L^20^, we firstly tried to determine glucosamine with a photometric kit (Megazyme, Wicklow, Ireland). Unfortunately, the high glucose concentration in the media, which is a prerequisite for enhanced organic acid excretion, led to a high background signal. To circumvent this, the samples had to be diluted at least 1:300, which in turn increased the detection limit from 1.3 mg/L to approximately 400 mg/L. Even if glucosamine had been present in the reported high extracellular concentrations, we would have been unable to detect any glucosamine with this photometric kit in the diluted samples.

Our next approach was based on an article^27^ reporting the use of glucosamine to quantify 6-aminopenicillanic acid. We reversed this method and used 6-aminopenicillanic acid to quantify glucosamine. This assay showed (i) no interferences with media components and (ii) its linear range was from 0.1 to 24 g/L glucosamine with a limit of detection (LOD) of 100 mg/L. But a major drawback of this method was the unknown specificity.

Summarising, both photometric tests were not satisfactory. To apply a different detection principle we turned to HPLC. The main focus of this investigation was laid on the determination of glucosamine, but as sugar alcohols such as erythritol, arabitol and mannitol were excreted under similar conditions^28,29^, method development began with a mixture of these four compounds. In this respect, two facts had to be considered, (i) the analytes possess no chromophore, thus detection methods other than UV/VIS had to be used, and (ii) all compounds of interest are very polar, so that sufficient retention on reversed phase material was difficult to achieve.

An optimum solution of the above mentioned problems was the use of hydrophobic interaction liquid chromatography (HILIC) material as stationary phase and evaporative light scattering detection (ELSD) and mass spectrometry (MS) for detection. HILIC is a more recent development in analytical science. It is a modification of normal phase chromatography in which hydrophilic stationary phases are used in combination with solvents of the reversed phase type^30^. Typically the separation begins with a high percentage of acetonitrile and during the analysis the amount of water is increased to enable elution of more polar compounds. HILIC is particularly suitable for the separation of polar compounds that cannot be retained on RP-material. ELSD and MS have been selected because their detection principles are not based on absorption; in addition, high resolution MS is a sensitive and specific detection mode that also enables identification of substances.

**Figure 2:**
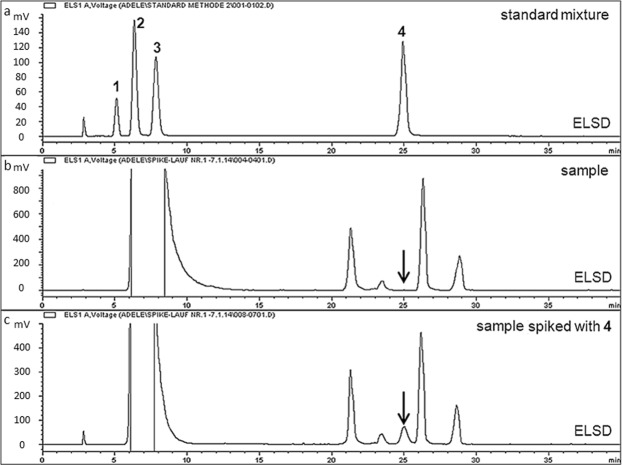
HPLC-ELSD analysis of (**a**) a standard mixture of erythritol (1), arabitol (2), mannitol (3) and glucosamine (4), (**b**) a representative sample, (**c**) a representative sample spiked with glucosamine.

The best possible separation of all four standards and of a typical sample solution by HPLC-ELSD is shown in Fig. 2. These chromatograms were accomplished after evaluating the influence of the HILIC material used (Poroshell 120 HILIC 2.7 µm, Atlantis silica HILIC 100 Å 3 µm and ZIC-HILIC 3.5 µm were also tested), the impact of mobile phase composition, its pH and the solvent gradient. As can be seen in Fig. 2a, the baseline separation of compounds **1** to **4** (**1**, erythritol; **2**, arabitol; **3**, mannitol; **4**, glucosamine) was possible in 25 min. When culture filtrates were analysed (Fig. 2b), a longer solvent gradient had to be applied to resolve compound **4** from substances of similar polarity. It was observed that **1** was missing in the samples and **2** and **3** were overlapped with a huge injection peak; therefore they could not be determined with the proposed method and were excluded from further investigation. A calibration curve (range 500 to 31 µg/mL) and the limit of detection (LOD) were determined for compound **4**. The regression equation for ELSD, being not a linear detector^31^, was found to be y = 0.0609 × ^1.6589^ (y = peak area, x = amount), with a correlation coefficient of R^2^ = 0.9989. The LOD of the method, defined as a signal to noise ratio of 3:1, was 7.8 µg/mL. This parameter was determined by the analysis of serial dilutions of **4**, but even with this method of analysis no signal corresponding to glucosamine (R_t_ = 25.0 min) was found in any of the culture filtrates analysed. The fact that the developed assay is well suited to resolve this compound from other matrix constituents was confirmed by spiking native culture filtrates with **4**. As exemplarily shown for a sample in Fig. 2c, an additional peak was clearly visible after spiking the native culture filtrate with **4**.

**Figure 3:**
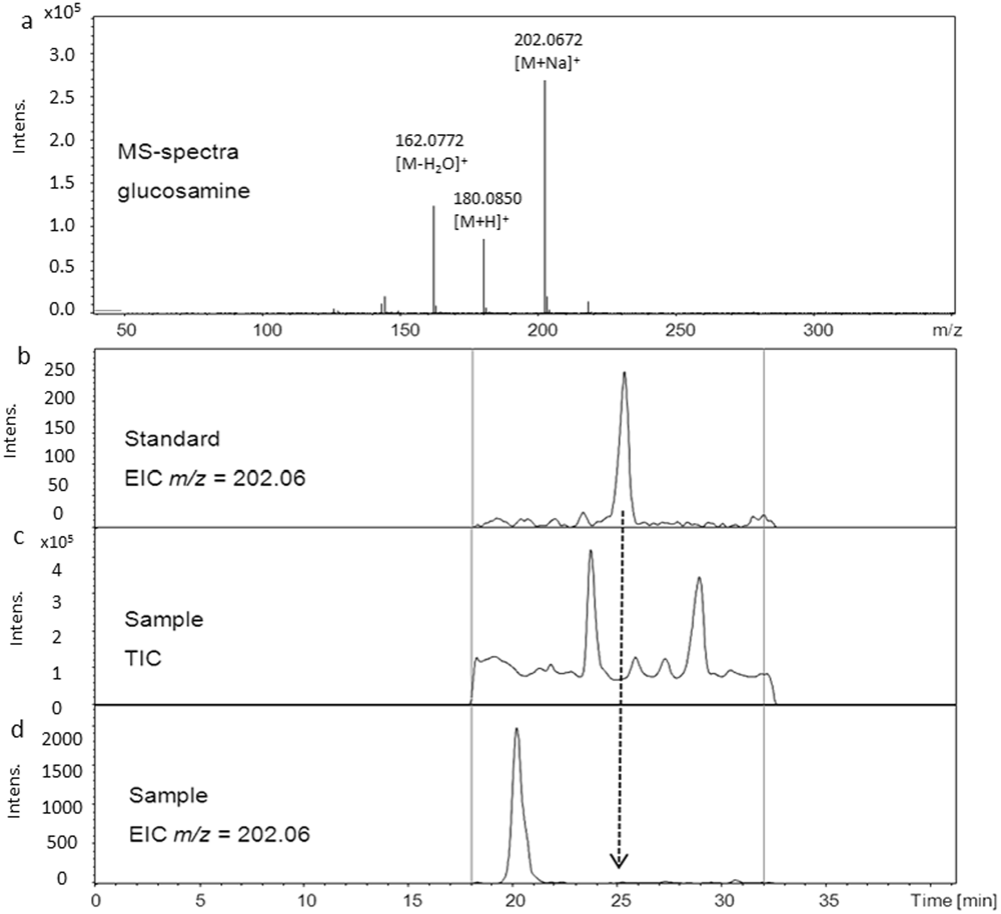
HPLC-MS analysis of (**a**) MS spectra of glucosamine; (**b**) the LC-MS analysis of a glucosamine standard (1 µg mL^−1^) in EIC mode; (**c**) LC-MS analysis of a *Penicillium ochrochloron* culture broth sample in TIC; (**d**) as well as EIC mode.

Possible limitations of using an ELSD are (i) its generally lower sensitivity compared to other detectors such as a diode array detector (DAD) and (ii) limited specificity. Both restrictions could be overcome by using a MS for detection instead. For this purpose, the HPLC instrument was coupled to a high-resolution time of flight (TOF) mass spectrometer. The LC conditions were the same as described above, only the mobile phase was introduced into the MS with a reduced volume (split ratio 1:2). Then the MS settings were optimised for the detection of glucosamine, and the LOD was determined to confirm sensitivity. In the most sensitive and selective EIC mode (extracted ion chromatogram) a LOD with 114 ng/mL was found. For the latter, only ions with an specific *m/z* value for the target analyte were selected, in this case corresponding to [M + Na]^+^ (202.06 ± 0.01; M_r_ of glucosamine is 179.17; Fig. 3a).

However, the analysis of culture broths of *A*. *niger* and *P*. *ochrochloron* bioreactor batches again showed no evidence for the presence of glucosamine. Although the compound could be easily detected in standards at low concentrations (Fig. 3b), it was not detected in any of the samples. Using a sample from an *A*. *niger* culture as a typical representative, small signals with retention times like glucosamine were observed in the total ion chromatogram (TIC) (Fig. 3c). In EIC mode, however, no agreement with **4** was possible with regard to both retention time and *m/z* value (Fig. 3d).

In summary, none of the analytical methods performed, whether photometric methods nor HPLC-ELSD or HPLC-MS, indicated the production of glucosamine by *A*. *niger* or *P*. *ochrochloron*. However, both organisms were found to excrete glycerol into the medium (Supplementary Fig. S2).

### Carbon balance for *P*. *ochrochloron* CBS123.824

Since *P*. *ochrochloron* is the organism of interest in our working group, we performed a carbon balance of ammonium limited bioreactor batch culture in duplicate. With this carbon balance we wanted to check whether or not a compound – other than glucosamine – was excreted in similar amounts as glucosamine^20^. Data from four different sampling points during the entire cultivation (Supplementary Fig. S3) were used: (1) the total carbon content at the beginning of cultivation at 0.3 h (=100%) was related to the carbon content during (2) the exponential growth phase (=95% of the initial carbon content) at 19.1 h and (3) shortly after the exhaustion of ammonium in the medium at 22.0 h (=104% of the initial carbon content), and (4) finally at the end of cultivation at 43.7 h (97% of the initial carbon content). The cultivation was terminated during the secondary growth phase before the culture reached the stationary phase (details see Supplementary Table S4 and Supplementary Figs S5–S7). These findings indicated that – if at all – only compounds in decidedly lower amounts than published for glucosamine excretion by *A*. *niger*^20^ were missing.

### Glucosamine as carbon and nitrogen source

**Figure 4:**
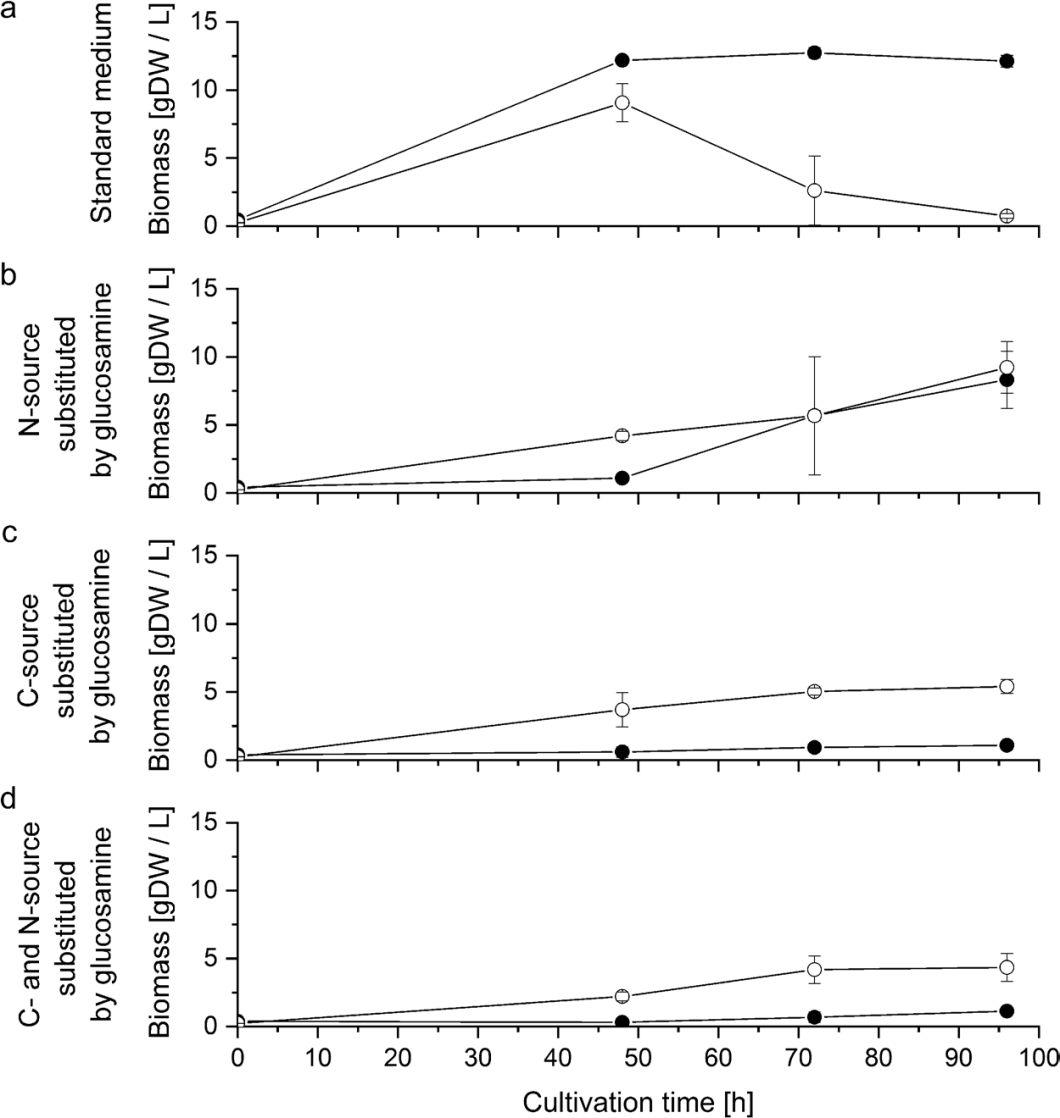
Growth of *P*. *ochrochloron* and *A*. *niger* in shake flask cultures without or with glucosamine substituting the C and/or N-source. (**a**) Standard medium (Supplementary Table S8), no substitution, (**b**) N-source is substituted by glucosamine, (**c**) C-source is substituted by glucosamine; (**d**) both C- and N-source are substituted by glucosamine. (●) *A*. *niger*, (○) *P*. *ochrochloron*.

The ability to transport glucosamine across the fungal plasma membrane is a prerequisite for the excretion of this metabolite. Since we could not detect any glucosamine excretion, we tested whether both fungi could principally utilise glucosamine as carbon and/or nitrogen source. For this purpose, we tested four different experimental settings (Supplementary Table S8) in a series of shake-flask experiments. In the setting for control glucose served as carbon and ammonium as nitrogen source; in the second setting, ammonium was substituted by glucosamine in equimolar amounts as the sole nitrogen source; in the third setting, glucose was substituted by glucosamine in equimolar amounts as the sole carbon source, and in the fourth experimental setting, glucosamine substituted both glucose and ammonium. Both fungi produced similar amounts of biomass when glucosamine substituted ammonium (Fig. 4), but when glucosamine substituted either glucose, or glucose and ammonium, *P*. *ochrochloron* produced five times more biomass than *A*. *niger* (Fig. 4).

## Discussion

The main objective of this work was to explore glucosamine excretion in two model filamentous fungi, i.e. *A*. *niger* and *P*. *ochrochloron*. Furthermore we studied the subsequent re-uptake of glucosamine in the early phases of organic acid excretion, as suggested by a previously published report for *A*. *niger* PM1^20^. For this investigation we performed a series of bioreactor batch experiments with *P*. *ochrochloron* CBS123.824 and *A*. *niger* CBS120.49. In addition, we also compared the published growth conditions for *A*. *niger*^20^ with our own standard growth conditions, which are known to trigger organic acid excretion in *P*. *ochrochloron*^26^. Surprisingly, despite similar growth characteristics (Table 2) we could not detect any glucosamine excretion in any of the experiments performed, even though we used four analytical methods with different sensitivity and specificity. A carbon balance for *P*. *ochrochloron* also revealed that no metabolite in amounts published for glucosamine with *A*. *niger*^20^ was missing in the periods of interest, especially in the early stage of citric acid excretion (Supplementary Table S4 and Supplementary Figs S5–S7).

This raises the question, why the observations on glucosamine production of *A*. *niger* PM1^20^ and *A*. *niger* CBS120.49 (present work) are this contradictory. Since the excretion of glucosamine in filamentous fungi is of high biotechnological interest – especially in the earlier reported amounts^20^ – this issue deserves a closer look. Although it cannot be excluded that these two *A*. *niger* strains show a different metabolite spectrum under identical cultivation conditions, several indications suggest that this strain specificity is rather unlikely.

The first evidence is the amount of ammonium in the culture medium (i.e. 38 mmol/L; Table 2), which actually does not support production of 48 g of glucosamine per liter^20^ (i.e. 268 mmol/L NH_4_). Indeed, considering the published biomass concentration^20^ and the general stoichiometric biomass formula of CH_1.8_O_0.5_N_0.2_, it is highly probable that approximately half of the available ammonium was already required for biomass formation during the early stages of citric acid excretion. Consequently, only about 20 mmol/L ammonium would have been available for glucosamine formation.

Secondly, the analysis of the first 30 h of cultivation with *A*. *niger* PM1 (Figs 5, 6, and 8 of the previously published report^20^) reveals that glucose uptake rates must be in the order of 2 mmol (g dry weight)/h. While these glucose-uptake rates under these conditions^32^ are typical for filamentous fungi, this rate is insufficient to cover the carbon demand for the proposed high amount of glucosamine excretion.

Last but not least, there are indications that the analytical data may have been misinterpreted by the authors. Unlike stated, the applied analytical system, a Sedex 55, is not a mass detector but an ELSD (Evaporative Light Scattering Detector). This type of detector is not specific and does not provide any compound characteristics such as *m/z* values, UV-spectra, etc. Thus, it was not certain whether the assigned signal was really glucosamine or a mixture of several compounds. By a more detailed examination, the signal associated to “glucosamine” in the chromatogram of the aforementioned report^20^ may have been the injection peak containing the target analyte (if any) and other substances.

So is fungal glucosamine production by excretion in an industrial scale feasible at all? Assuming that extracellular glucosamine is not produced by a cell wall bound enzyme, its excretion must involve transmembrane transport. The experimental data in this work support the assumption that these two fungi can transport glucosamine across their plasma membrane: Both, *A*. *niger* and *P*. *ochrochloron* were able to use glucosamine as C and/or N-source (Fig. 4). This transmembrane transport is one prerequisite for using these organisms for the production of glucosamine in an economically attractive way. However, most likely similar to bacteria^5,10^ metabolic engineering might be the key to achieve this goal.

## Methods

### Organisms and growth conditions

#### Organisms

*P*. *ochrochloron* CBS123.824 is a strain isolated from a heavy metal contaminated soil by one of the authors (W. B.) and subcultivated in our laboratory since 1986. *A*. *niger* A158 (=CBS120.49) is a citric acid producing strain that has been reported to excrete glucosamine^25^. This *A*. *niger* strain was already used in an earlier study^33^ by one of the authors (W. B.).

#### Bioreactor precultures

Conidia suspensions of both fungi were produced as described earlier^33^ on sterilised rye. Preculture of *P*. *ochrochloron* used a Hepes-glucose medium^34^. Precultures of *A*. *niger* were performed as earlier described^20^ with one modification: The original glucose concentration of 749 mM was reduced to 250 mM, as this lower concentration was also reported to trigger glucosamine excretion^20^. All precultures were cultivated in 500 mL Erlenmeyer flasks with 100 mL medium and an inoculation density of 10^6^–10^7^ conidia per mL. Further incubation conditions were according to the standard protocols described earlier^20,34^.

#### Bioreactor batch cultivation and sampling

All bioreactor batch experiments were performed either in a BIOSTAT M (Braun/Sartorius, Germany) or a KLF 2000 (Bioengineering, Switzerland) bioreactor with a working volume of 1.8 L. The standard equipment was described in previous studies^26,32,35^. According to the report about *A*. *niger* PM1^20^, media and culture conditions (e.g. inoculum volume, temperature, pH, stirring speed) were applied to study glucosamine excretion both in *P*. *ochrochloron* and *A*. *niger*. In addition, *P*. *ochrochloron* was also grown under our standard ammonium limited growth conditions at pH 7 as described earlier^26^. All cultivations were performed at least in duplicates. The bioreactor batch experiments were sampled regularly in the following way: After discarding the waste volume, 5 mL culture broth was immediately vacuum filtered through a 0.45 µm cellulose acetate filter. Aliquots of the filtrate were instantly frozen at −40 °C and stored at −20 °C until further analysis. The biomass on the filter was washed with 60 mL distilled water, dried at 105 °C for at least 12 hours, brought to room temperature in an exsiccator and weighed.

#### Growth on glucosamine as C and/or N source in shake flask experiments

Cultures of *A*. *niger* and *P*. *ochrochloron* were performed with four replicates in 100 mL Erlenmeyer shake flasks with four different media (Supplementary Table S8). Solutions with glucose and salts were autoclaved separately and mixed under sterile conditions after reaching room temperature. Sterilised trace element solution^26^ (cellulose acetate filter; 0.22 µm) was added (0.5 mL/L). Shake flasks were filled with 20 mL medium and inoculated with 0.2 mL of a conidial suspension (10^8^ conidia per mL), resulting in a final concentration of 10^6^ conidia per mL. The cultures were incubated on a rotary shaker with 150 rpm at 30 °C. Samples were taken at 0 h, 48 h, 72 h and 96 h of cultivation time. The dry weight of the biomass was determined after membrane filtration (cellulose acetate; 0.45 µm) following the procedure mentioned before. The pH, concentrations of glucose, ammonium, glucosamine, and organic acids were determined in the filtrate as follows.

### Analytics

#### Chemicals

All standard compounds used (glucosamine, erythritol, arabitol and mannitol; purity ≥98%) as well as chemicals (acetic acid, ammonium acetate, 6-aminopenicillanic acid, etc.) and solvents (acetonitrile) were purchased from Sigma (St. Louis, MO, USA) or Merck (Darmstadt, Germany). HPLC-grade water for analysis was obtained from an Arium water purification system (Sartorius, Göttingen, Germany).

#### Analysis of main media components and excreted metabolites

Test kits from Megazyme International (Wicklow, Ireland) were used to determine D-glucose (K-GLUC, GOPOD format), D-glucosamine (K-GAMIN), glycerol (K-GCROL), gluconic acid (K-GATE) and citric acid (K-CITR). A second photometric test to quantify glucosamine was performed by reversing the 6-aminopenicillanic acid reaction of a previously published method^27^. Residual nutrient concentrations in the medium of ammonium^36^ and phosphate^37^ were measured in macroassays (U-2001 UV/VIS spectrophotometer (Hitachi) or in microtiter plates (Sunrise Plate Reader; Tecan). For all photometric assays, calibration curves were established in the respective growth media to detect possible interferences. Pyruvate, citrate, succinate, fumarate, malate and oxalate were determined by isocratic HPLC using an Aminex HPX 87 H column (Bio-Rad)^38^.

#### HPLC-ELSD and HPLC-MS analysis of glucosamine

The separations were performed on an Agilent 1200 HPLC system (Santa Clara, CA, USA) equipped with vacuum degasser, binary pump, autosampler, column oven and DAD detector. The optimal stationary phase was a Kinetex HILIC 100 Å column (150 × 4.6 mm, 2.6 µm particle size; Phenomenex, Torrance, CA, USA) operating in gradient elution mode. For this purpose, the mobile phase included acetonitrile (A) and an aqueous 20 mM ammonium acetate solution with a pH of 3.0 (B); the pH was adjusted with glacial acetic acid. The gradient applied was 94A/6B in 40 min to 70A/30B, followed by a wash (10 min with 30A/70B) and an equilibration step (10 min with 94A/6B). Flow rate, column temperature and injected sample volume were 0.7 mL/min, 25 °C and 10 µL, respectively. Either an ELSD (Sedex 85 LT-ELSD; Sedere, Alfortville, France) or a microTOF-QII mass spectrometer (Bruker, Bremen, Germany) was used for detection. The ELSD settings were nebuliser temperature 60 °C, nebuliser pressure 4.3 bar (nitrogen) and gain 10. The MS instrument was operated in positive ESI mode, with the nebuliser set to 1.6 bar (nitrogen), dry gas at 8 L/min (nitrogen), and a dry temperature of 200 °C. The capillary voltage was set to −3.5 kV and the m/z values were recorded from 50 to 500.

Aqueous solutions of standards (concentration 0.5 mg/mL) for method development were prepared fresh daily. These aqueous solutions were used for calibration and determination of LOD. Fungal culture samples of *A*. *niger* and *P*. *ochrochloron* were analysed natively, i.e. culture broths were taken directly from the bioreactor, membrane-filtered (cellulose acetate; 0.45 µm) and frozen at −20 °C. The samples were then analysed by HPLC. For HPLC analysis the samples were thawed and immediately examined without further treatment.

### Carbon balance

A carbon balance was established using two methods. First, the total carbon was calculated from carbon in biomass (determined with a C/N analyser) plus carbon in glucose and excreted metabolites plus carbon excreted as carbon dioxide. Secondly, the total carbon was calculated from carbon in biomass plus carbon in culture filtrate (determined with a C/N analyser) plus carbon excreted as carbon dioxide. A carbon balance was calculated for two *P*. *ochrochloron* cultivations and four time points: 0.3 h (which was assumed to be 100%), and then 19.1 h, 22.0 h and 43.7 h (Supplementary Fig. S3).

## Supplementary information

Supplementary Informations

## Data Availability

All data generated or analysed during this study are included in this article (and its Supplementary Information files).
