# Supplementary material for: Critical evaluation of a putative glucosamine excretion by Aspergillus niger CBS120.49 and Penicillium ochrochloron CBS123.824 under citric acid producing conditions
Source: Sci Rep. 2019 May 16;9:7496. doi: 10.1038/s41598-019-43976-z (PMC6522597; doi:10.1038/s41598-019-43976-z)
Supplement: Supplementary file 1 — Supplementary Informations [file 41598_2019_43976_MOESM1_ESM.pdf]

Supplementary Materials to the article:

Critical evaluation of a putative glucosamine excretion by *Aspergillus niger* CBS120.49 and *Penicillium ochrochloron* CBS123.824 under citric acid producing condition

Desirée Josefine Artmann[1 *], Werner Amrain[1], Adele Murauer[2], Markus Ganzera[2], Pamela Vrabl[1], Christoph Walter Schinagl[1], Wolfgang Burgstaller[1]

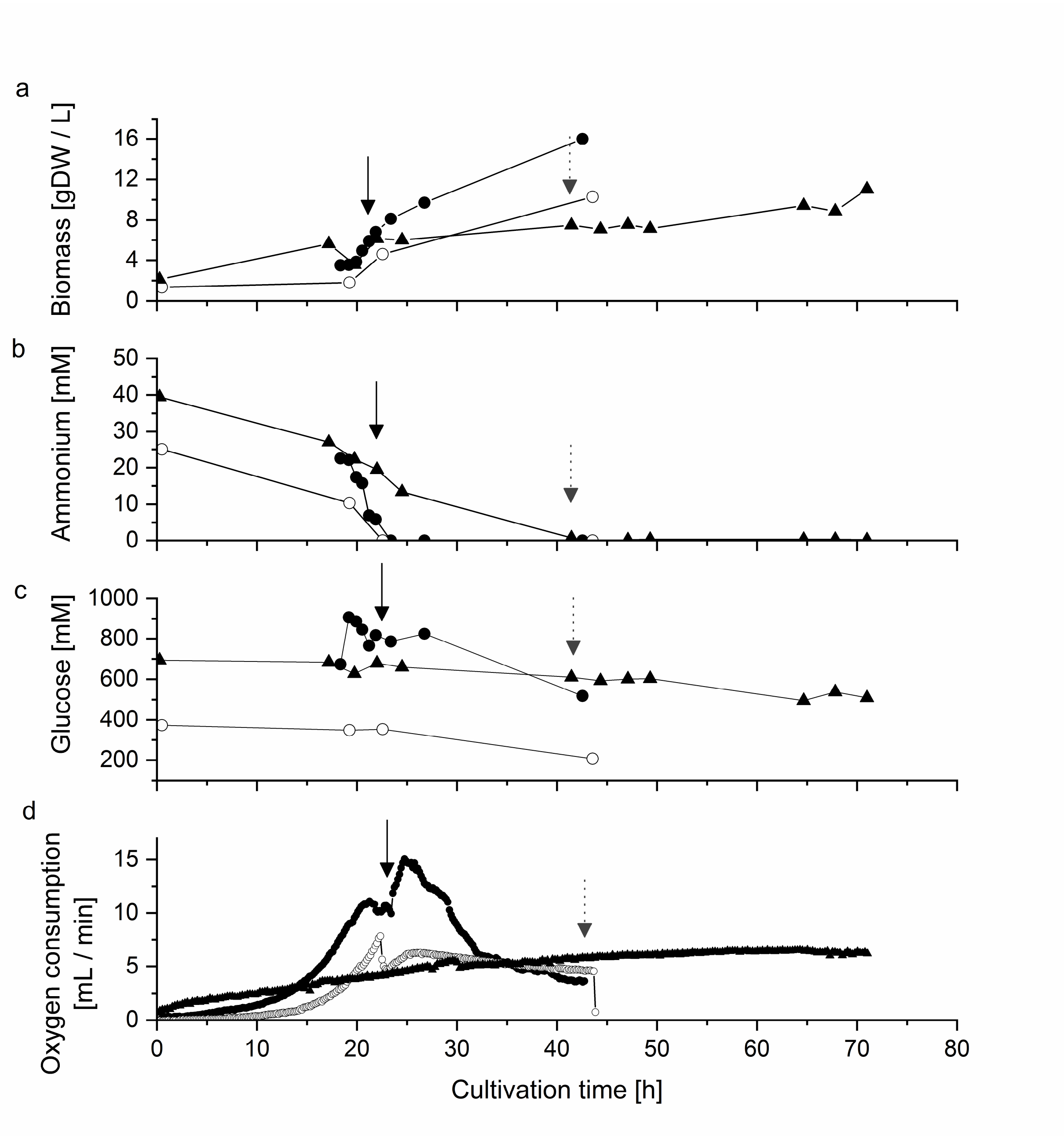

Supplementary Figure S1: Growth characteristics of the duplicate bioreactor batch cultures of *Penicillium ochrochloron* CBS 123.824 and *Aspergillus niger* CBS 120.49 depicted in Figure 1. (**a**) Biomass formation, (**b**) ammonium concentration, (**c**) glucose concentration and (**d**) oxygen consumption. Black arrows denote the time point of ammonium exhaustion in both *P. ochrochloron* cultures, dashed grey arrows in *A. niger* cultures. (▲) *A. niger*, cultivation conditions after reference 20. (●) *P. ochrochloron* cultivation conditions after reference 20. (○) *P. ochrochloron* in ammonium limited standard medium after reference 26.

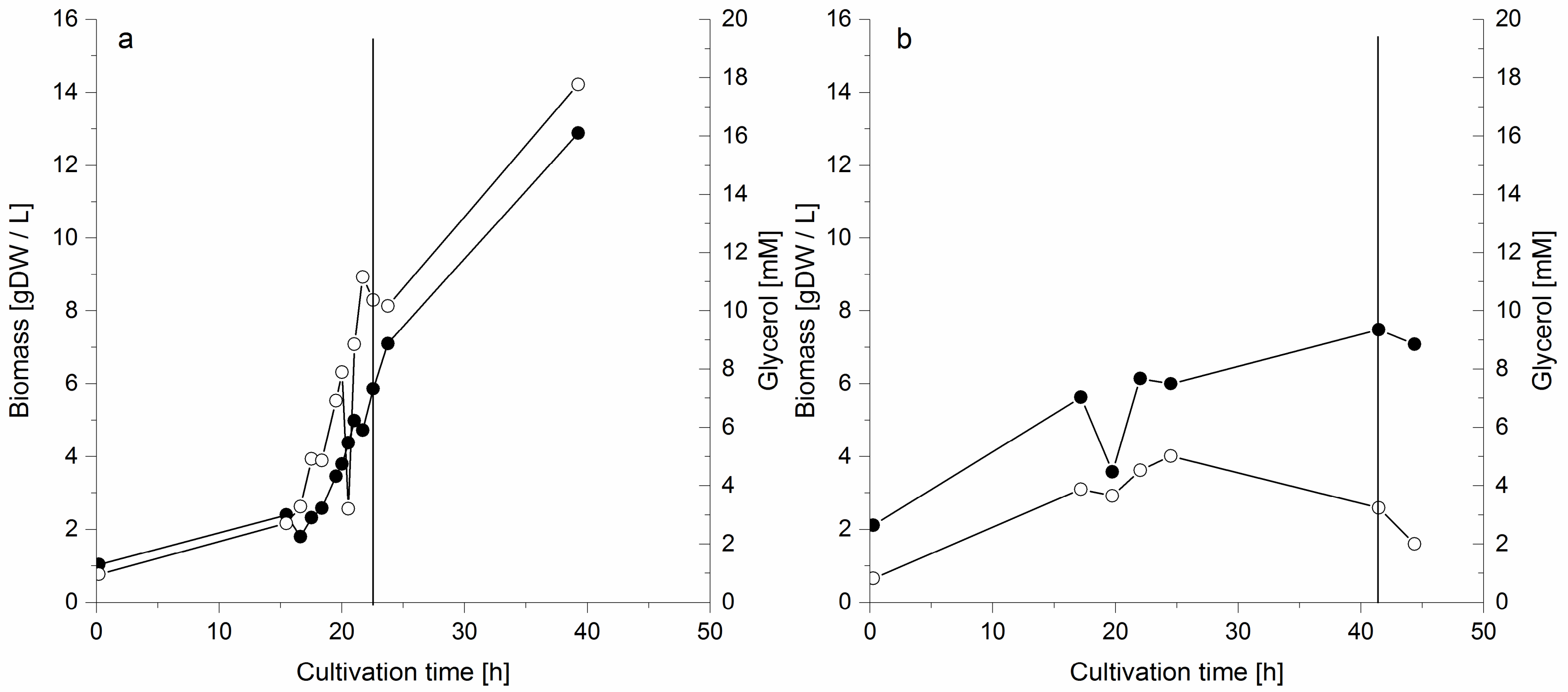

Supplementary Figure S2: Extracellular glycerol concentration and biomass concentration of (a) *P. ochrochloron* and (b) *A. niger* batch cultures. The vertical line indicates ammonium depletion in the medium. (●) biomass, (○) glycerol.

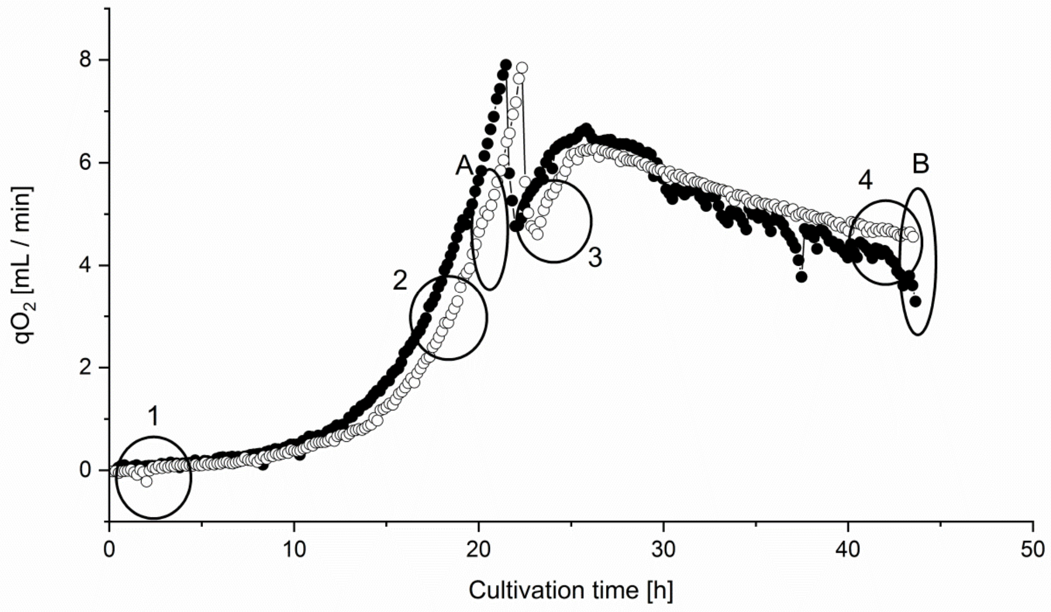

Supplementary Figure S3: Oxygen consumption curves and sampling time points to calculate the carbon balance of duplicate *P. ochrochloron* batch cultures. (●) Experiment 1 and (○) Experiment 2. A, B = C/N sample (100 mL), 1-4 = normal sample (6-7 mL). Details for the estimation of the carbon balance see Supplementary Table S4.

Supplementary Table S4: Detailed carbon balance for duplicate bioreactor batch cultures of *P. ochrochloron*. Bold written values are sampling points A and B (Supplementary Figure S3), normal written values are sampling points 1 to 4 (Supplementary Figure S3).

	**Cultivation time [h]**	Carbon g/L stored in / lost by								Total detected C	
		**filtrate**	**biomass**	**glucose**	**glycerol**	**TCAs**	**glucosamine**	**gluconic acid**	**carbon dioxide**	**g/L**	**%**
BM N° 148	0.3		0.31	27.36	0.11	0.01	0	0	0	27.8	100
	19.1		1.13	24.58	0.29	0.02	0	0	0.33	26.36	94.83
	**19.4**	**30.75**	**1.20**						**0.35**	**32.29**	**116.17**
	22		2.05	25.51	0.62	0.06	0	0	0.69	28.93	104.08
	43.7		6.74	16.47	0.70	0.66	0	0	3.87	28.44	102.32
	**43.8**	**20.31**	**6.74**						**3.89**	**30.93**	**111.27**
BM N° 149	0.5		0.63	26.88	0.14	0.02	0	0	0	27.66	100
	19.3		0.84	25.06	0.23	0.04	0	0	0.25	26.43	95.53
	**20.4**	**27.81**	**1.17**						**0.32**	**29.3**	**105.9**
	22.6		2.15	25.43	0.38	0.05	0	0	0.59	28.6	103.39
	43.6		5.70	14.89	0.46	0.74	0	0	3.75	25.54	92.32
	**43.7**	**14.49**	**5.70**						**3.77**	**23.97**	**86.63**

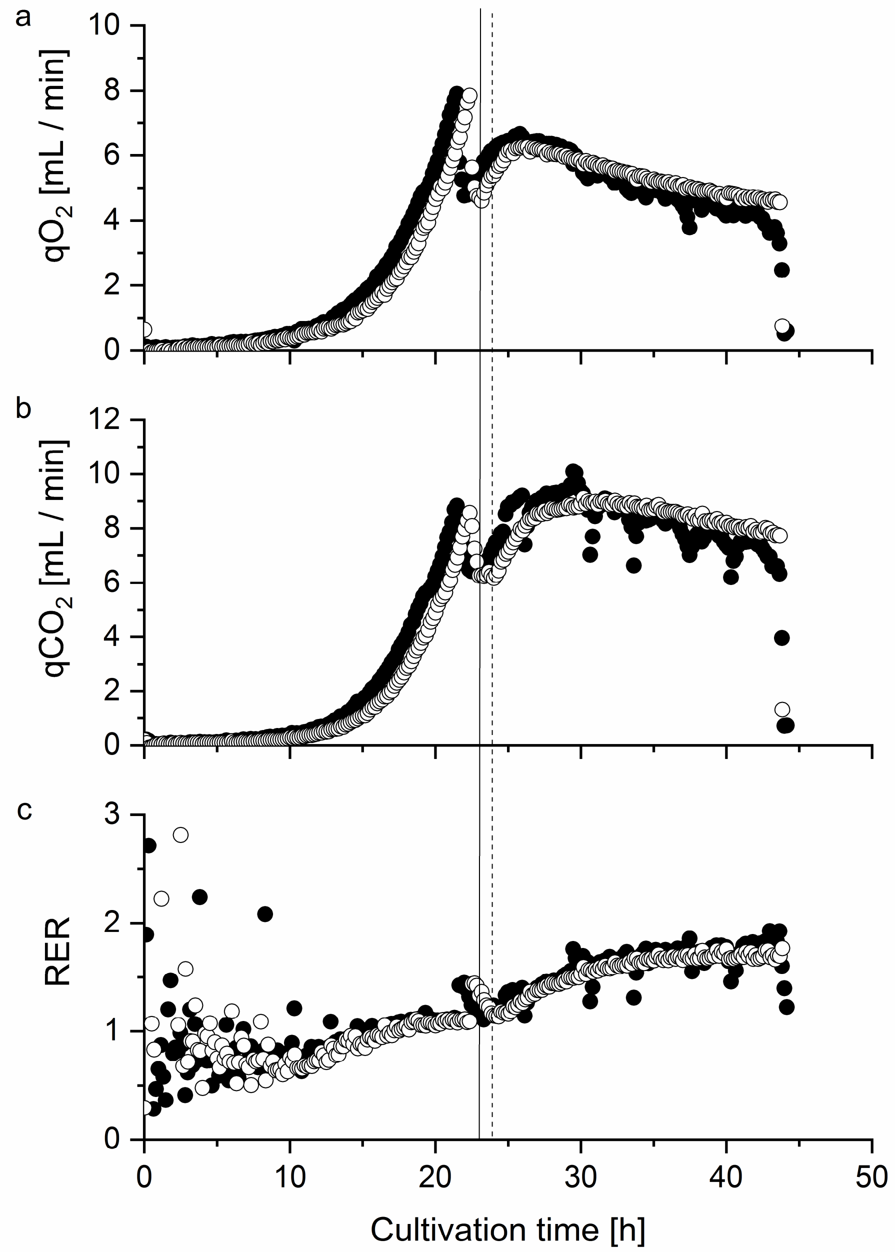

Supplementary Figure S5: Oxygen consumption, carbondioxide evolution and respiratory quotient of duplicate *P. ochrochloron* batch cultures used for carbon balance (see Supplementary Table S4). (●) Experiment 1 and (○) Experiment 2. (**a**) Oxygen consumption qO2; (**b**) Carbondioxide evolution qCO_2; (**c**) Respiratory quotient RER; The vertical lines (bold for experiment 1 and dashed for experiment 2) indicate the depletion of ammonium in the medium.

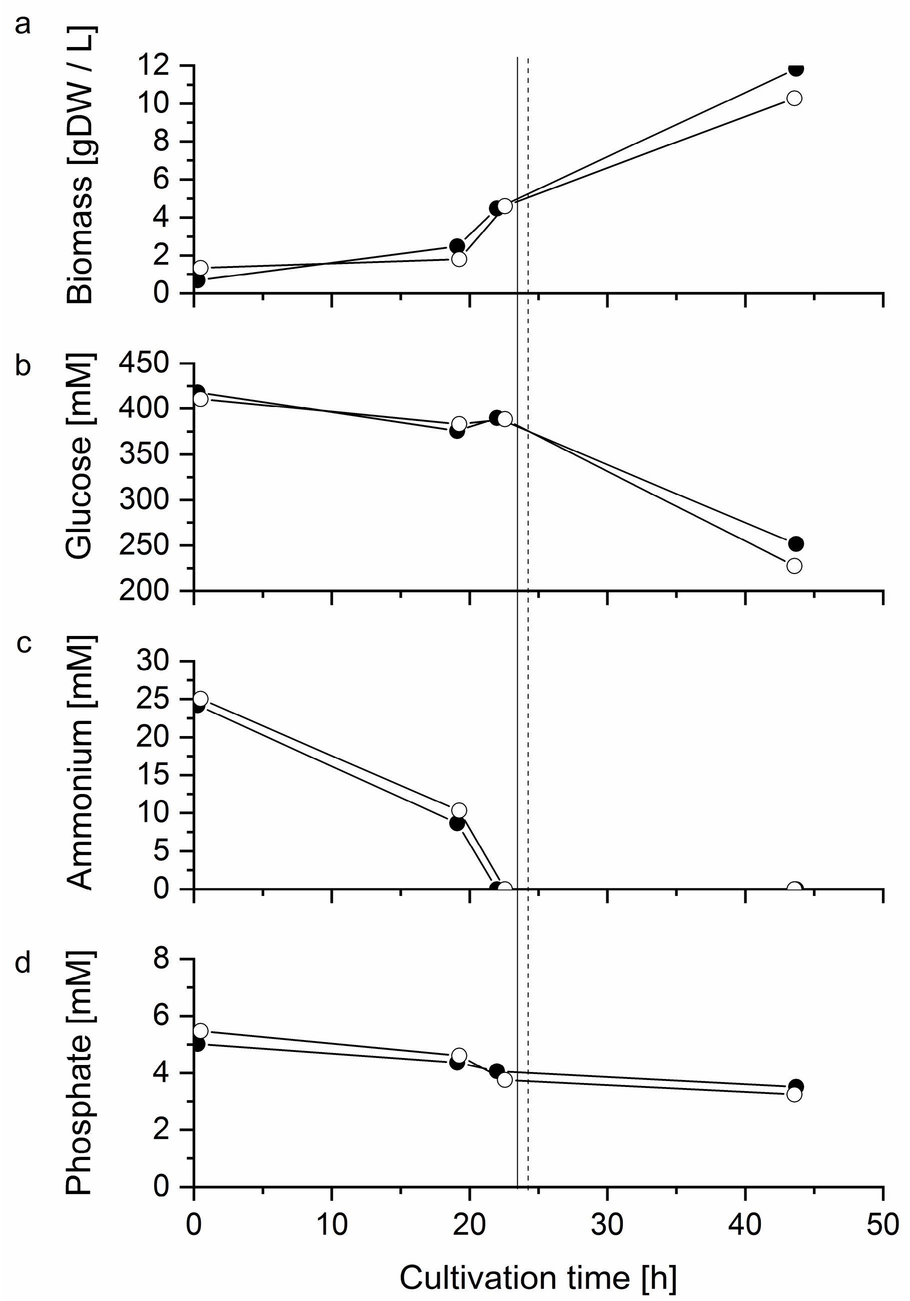

Supplementary Figure S6: Biomass production and residual nutrient concentrations of duplicate *P. ochrochloron* batch cultures used for carbon balance (see Supplementary Table S4). (●) Experiment 1 and (○) Experiment 2. **a**) biomass concentration; (**b**) glucose; (**c**) ammonium; (**d**) phosphate. The vertical lines (bold for experiment 1 and dashed for experiment 2) indicate the depletion of ammonium in the medium.

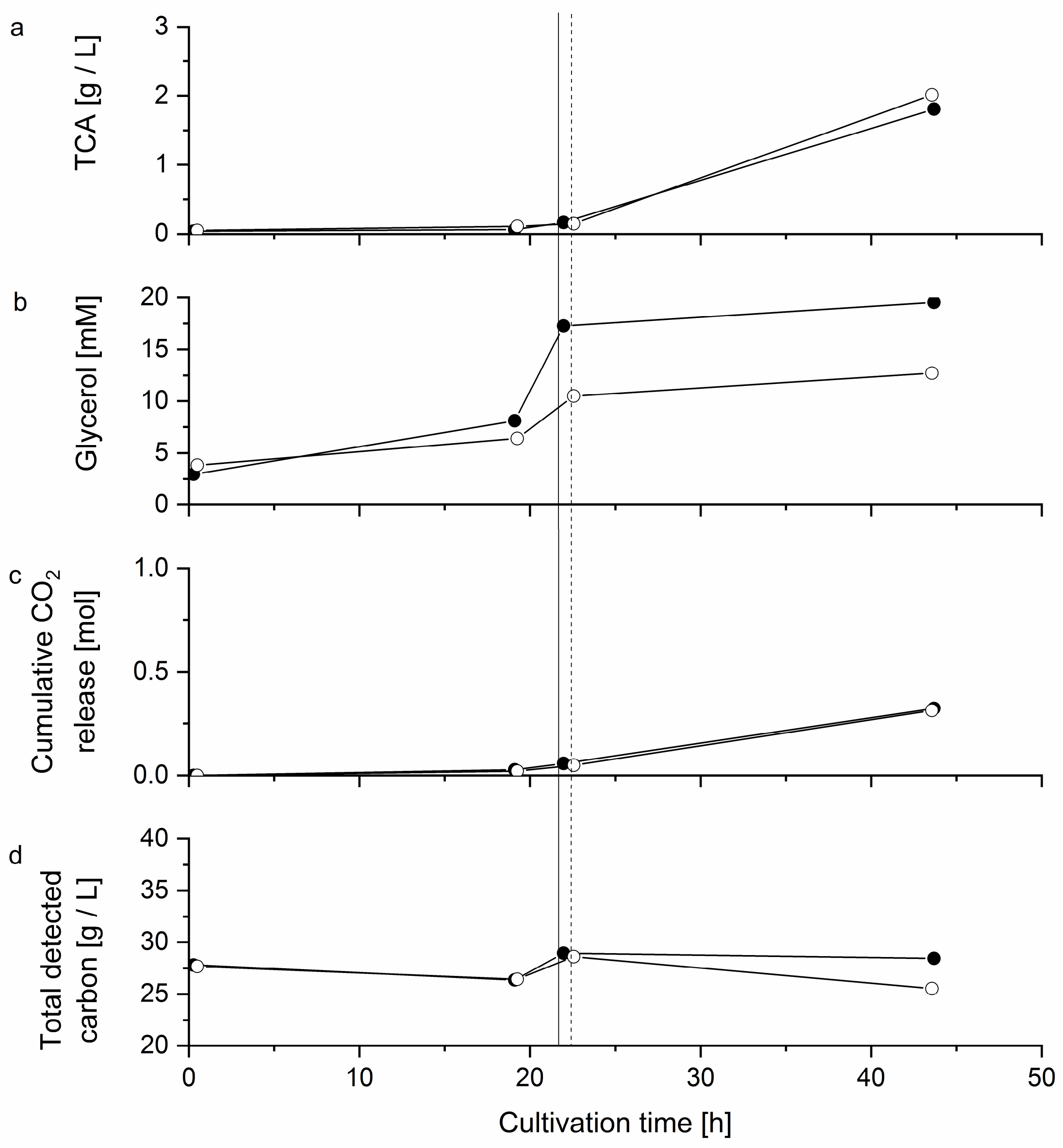

Supplementary Figure S7: Organic acid and glycerol excretion, cumulative carbon dioxide release and total detected carbon of duplicate *P. ochrochloron* batch cultures used for carbon balance (see Supplementary Table S4). (●) Experiment 1 and (○) Experiment 2. **a**) the total concentration of the TCA; (**b**) glycerol; (**c**) cumulative CO_2 emission; (**d**) total detected carbon. The vertical lines (bold for experiment 1 and dashed for experiment 2) indicate the depletion of ammonium in the medium.

Supplementary Table S8: Composition of the different media used for the glucosamine substitution experiments.

**Medium**	**Standard**	**Substituted N-source**	**Substituted C-source**	**Substituted C- and N-source**
Substance	**[g / L]**	**[g / L]**	**[g / L]**	**[g / L]**
Glucose x H_2O	50	50	0	0
Glucosamine x HCl	0	8	54	54
$(NH_4)_2SO_4$	2.5	0	2.5	0
KH_2PO_4	2	2	2	2
$MgSO_4$ x $7H_2O$	0.5	0.5	0.5	0.5
Trace element solution[26]	10 mL / L	10 mL / L	10 mL / L	10 mL / L
